# Placenta microstructure and microcirculation imaging with diffusion MRI

**DOI:** 10.1002/mrm.27036

**Published:** 2017-12-11

**Authors:** Paddy J. Slator, Jana Hutter, Laura McCabe, Ana Dos Santos Gomes, Anthony N. Price, Eleftheria Panagiotaki, Mary A. Rutherford, Joseph V. Hajnal, Daniel C. Alexander

**Affiliations:** ^1^ Centre for Medical Image Computing and Department of Computer Science University College London London UK; ^2^ Centre for the Developing Brain, King's College London London UK; ^3^ Biomedical Engineering Department King's College London London UK

**Keywords:** placenta, diffusion MRI, microstructure, intravoxel incoherent motion, model selection, Bayesian information criterion

## Abstract

**Purpose:**

To assess which microstructural models best explain the diffusion‐weighted MRI signal in the human placenta.

**Methods:**

The placentas of nine healthy pregnant subjects were scanned with a multishell, multidirectional diffusion protocol at 3T. A range of multicompartment biophysical models were fit to the data, and ranked using the Bayesian information criterion.

**Results:**

Anisotropic extensions to the intravoxel incoherent motion model, which consider the effect of coherent orientation in both microvascular structure and tissue microstructure, consistently had the lowest Bayesian information criterion values. Model parameter maps and model selection results were consistent with the physiology of the placenta and surrounding tissue.

**Conclusion:**

Anisotropic intravoxel incoherent motion models explain the placental diffusion signal better than apparent diffusion coefficient, intravoxel incoherent motion, and diffusion tensor models, in information theoretic terms, when using this protocol. Future work will aim to determine if model‐derived parameters are sensitive to placental pathologies associated with disorders, such as fetal growth restriction and early‐onset pre‐eclampsia. Magn Reson Med 80:756–766, 2018. © 2017 The Authors Magnetic Resonance in Medicine published by Wiley Periodicals, Inc. on behalf of International Society for Magnetic Resonance in Medicine. This is an open access article under the terms of the Creative Commons Attribution License, which permits use, distribution and reproduction in any medium, provided the original work is properly cited.

## INTRODUCTION

The placenta is a vitally important yet understudied organ 1. Abnormalities in the microscopic and macroscopic anatomy can disrupt the flow of blood, and therefore the transfer of oxygen and nutrients from mother to fetus. These effects are associated with major pregnancy complications such as fetal growth restriction (FGR) 2, 3, 4, and early onset pre‐eclampsia 5, 6. FGR affects 5–10% of all pregnancies 2; pre‐eclampsia affects 2–8%, and both increase with risk factors such as obesity 7, 8, 9, 10. However these abnormalities are difficult to detect before the onset of symptoms. For example, diagnosis and monitoring of FGR is currently limited to measuring fetal biometry and heart rate, amniotic fluid volume, and assessment of blood flow using Doppler ultrasound of the umbilical cord and uterine arteries 11. However, at the point of diagnosis with Doppler ultrasound there has already been substantial inhibition of placental function, and damage to placental microstructure. Clinicians aim to identify FGR as early as possible, so that the fetus can be closely monitored and the delivery planned accordingly 12. Early diagnosis is also vital for the management of pre‐eclampsia 13.

Imaging techniques capable of assessing early placental development could offer an important new window for the earlier detection of pregnancy complications. Development of non‐invasive, in vivo techniques for measuring blood flow (beyond Doppler ultrasound) and oxygenation is an active field of research. For example, maternal blood oxygenation and maternal blood flow have been quantified with blood‐oxygen‐level dependent MRI 14, 15 and dynamic contrast‐enhanced MRI 16, respectively. Diffusion‐weighted MRI (DWI) is also emerging as a promising technique for quantifying placental function 17, 18, but previous studies are limited to standard simple diffusion models, such as apparent diffusion coefficient (ADC) mapping, diffusion tensor imaging (DTI) 19, and intravoxel incoherent motion (IVIM) 20.

In this paper, we assess a variety of novel mathematical models of the diffusion MRI signal from in vivo human placenta at 3T. In addition to ADC, DTI and IVIM we consider a range of multicompartment models with the potential to provide additional information on tissue structure and function. The aim is to find the best models to underpin diffusion‐based microstructure imaging techniques to provide new information, and hence enable earlier detection of placental abnormalities.

## METHODS

### Placenta Structure

**Figure 1:**
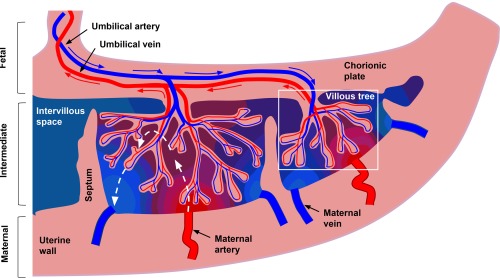
Schematic representation of blood flow through the placenta and surrounding tissue. Blue and red arrows show the flow directions of oxygenated (red) and deoxygenated (blue) fetal blood through the placental vasculature. For clarity, only the largest villi are included (for normal placentas terminal villi make up 40% of villous tree volume [21]). Dashed white arrows show idealized flow lines through intervillous space for maternal blood. Idealized oxygenation states are represented by the red to blue color gradient.

The placenta is a highly vascular organ, consisting of 15–40 cotyledons separated by septa. Each cotyledon contains one or more functional units, which usually consist of a paired spiral artery and fetal villous tree. Figure 1 summarizes the placental structure and principle routes of blood flow. There are two separate, non‐mixing compartments of blood: the intervillous space (where maternal blood resides) and the fetal vasculature (containing fetal blood). The flow of blood in these compartments has very different characteristics. Maternal blood flows slowly through the large pools of intervillous space, bathing the fetal villi and enabling oxygen exchange across the villous tree surface. On the other hand, fetal blood perfuses through a convoluted path of fetal vessels. Although the placenta is a highly vascular organ, it is immediately bounded by the uterine wall and chorionic plate, which contain trophoblastic cells, and various types of fibrous cells (21, p. 158).

### Diffusion Models

It follows that there are a wide variety of structures and processes which need to be considered in biophysical models of water diffusion in the placenta. First, we expect a slow‐attenuating DWI signal component from water diffusing within tissue, such as fetal vessel walls. We also expect that fetal blood within the convoluted fetal vasculature flows incoherently at the voxel scale, leading to a pseudo‐diffusion effect. This will contribute a fast‐attenuating component to the DWI signal. The characteristics of maternal blood flow are very different, and it is not immediately clear how these will affect the signal. Maternal blood enters the placenta through spiral arteries, flows through intervillous space, then returns to decidual veins in the uterine wall. Within vasculature, i.e., spiral arteries and decidual veins, maternal blood may exhibit fast incoherent flow on the voxel‐scale, similar to fetal blood. Therefore in uterine wall areas a fast‐attenuating component to the DWI signal is likely. On the other hand, coherent flow of maternal blood in the intervillous space of the placenta should cause little signal attenuation. However, we do expect signal attenuation due to diffusion within the flow, with a similar diffusivity to water at body temperature (3 × 10^−3^ mm^2^ s^−1^). Additionally, flow through highly convoluted spaces proximal to fetal villi may appear incoherent at the MRI voxel scale, leading to a pseudo‐diffusion effect like microcirculatory perfusion. We would expect this component to have pseudo‐diffusivity higher than 3 × 10^−3^ mm^2^ s^−1^—since the maternal blood transit time through intervillous space is estimated at 25 s 40—but lower than that induced by microcirculation. This idea is consistent with a study in mice, where the estimated maternal blood ADC in the placenta was $3.1\;\pm \;0.4\times 10^{-3}$ mm^2^ s^−1^ 22.

Considering these observations about placental structure and microstructure, we constructed a set of 14 plausible diffusion models. The models are summarized in Table 1; they are named following the terminology of Ref. 23. All models are multicompartment combinations of the following: ball, stick, zeppelin, tensor, sphere; and they all assume no water exchange between compartments. The “ball” compartment models isotropic diffusion (i.e., an ADC model). The “stick” compartment is maximally anisotropic, assuming that water diffuses only in a single direction; the signal is given by $S=\exp\left(-bD_{v}(n.G)\right)$ where n is the fiber direction and G is the gradient direction. A “tensor” models the signal using the full diffusion tensor, and “zeppelin” is a cylindrically symmetric tensor (as 23). Ball, stick, zeppelin, and tensor are therefore all special cases of the diffusion tensor model, i.e., a Gaussian displacement distribution, and do not explicitly model restriction. On the other hand the “sphere” compartment models water restricted in impermeable spheres. There are many possible combinations of these compartments that we have not included in the 14 models. We limit the set to those that are biologically plausible (e.g., we do not include models with a stick compartment for extracellular diffusion), and to those with a manageable number of parameters (e.g., excluding the 13‐parameter tensor‐tensor model).

**Table 1:** Summary of Multicompartment Models Fitted to the Diffusion‐Weighted MRI Signal. The Columns “Perfusion,” “Diffusion,” and “Restricted” Denote the Compartment Used to Model Each Contribution to the Signal. Columns are Merged When Contributions are Combined, e.g., Ball‐Ball Combines Separate Diffusion and Restricted Compartments into a Single “Diffusion” Compartment.

	Model compartments			
Model type	Perfusion	Diffusion	Restricted	Parameters
Single compartment	Ball			*D*_v_
	Stick			$D_{v},\phi ,\theta$
	Tensor			$D_{v}^{∥},D_{v_{1}}^{⊥},D_{v_{2}}^{⊥},\phi ,\theta ,\psi$
Iso‐iso	Ball	Ball		$D_{v},D,f_{v}$
	Ball		Sphere	$D_{v},D_{\text{sphere}},r,f_{\text{sphere}}$
	Ball	Ball	Sphere	$D_{v},D,D_{\text{sphere}},r,f_{v},f_{\text{sphere}}$
Aniso‐iso	Stick	Ball		$D_{v},D,\phi ,\theta ,f_{v}$
	Tensor	Ball		$D_{v}^{∥},D_{v_{1}}^{⊥},D_{v_{2}}^{⊥},\phi ,\theta ,\psi ,D,f_{v}$
	Zeppelin	Ball		$D_{v}^{∥},D_{v}^{⊥},\phi_{v},\theta_{v},D,f_{v}$
	Stick	Ball	Sphere	$D_{v},\phi ,\theta ,D,r,f_{v},f_{\text{sphere}}$
Iso‐aniso	Ball	Zeppelin		$D_{v},D^{∥},D^{⊥},\phi ,\theta ,f_{v}$
	Ball	Tensor		$D_{v},D^{∥},D_{1}^{⊥},D_{2}^{⊥},\phi ,\theta ,\psi ,f_{v}$
Aniso‐aniso	Stick	Zeppelin		$D_{v},\phi_{v},\theta_{v},D^{∥},D^{⊥},\theta ,\phi ,f_{v}$
	Zeppelin	Zeppelin		$D_{v}^{∥},D_{v}^{⊥},\phi_{v},\theta_{v},D^{∥},D^{⊥},\theta ,\phi ,f_{v}$

The motivation for compartment models is to capture the properties of distinct water pools. Here, we expect that perfusion compartments (i.e., those associated with fast‐attenuating signal components) capture blood (fetal or maternal) perfusing within vasculature. Diffusion compartments (associated with slow‐attenuating signal components) capture signal primarily from: (i) diffusion within tissue and (ii) diffusion within blood, but may also have a contribution from (iii) slow, incoherent flow of maternal blood. Despite the expectation for maternal blood perfusion to affect both the perfusion and diffusion compartments, we retain the labels “perfusion” and “diffusion,” as these remain the dominant effects we expect each to capture.

In classical IVIM (we refer to this specific model from now on as “ball‐ball”), perfusion and diffusion compartments are both treated as isotropic. However this may not hold in areas containing fibrous cells, or in areas where the vasculature has a coherent orientation, which is likely in the placenta. Therefore, we explore models which separately consider anisotropy in the fast‐ and slow‐attenuating signal components; we refer to this general class of models as “anisotropic IVIM.” We also consider further model refinements by splitting the diffusion compartment, yielding a three‐compartment model (similar to those used in cancer imaging, e.g., 24). Such models include a restricted compartment, which assumes that water is trapped in impermeable spheres, and an extra‐cellular extra‐vascular compartment which has been previously modeled with a diffusion tensor 24. We have therefore proposed extending ball‐ball (i.e., IVIM) in two ways: by allowing anisotropy in the diffusion and perfusion compartments (yielding “anisotropic IVIM” models), and also modeling the effect of diffusion restriction.

We also consider one‐compartment models as a baseline. In particular ADC and DTI models, which combine perfusion, diffusion and restriction contributions into a single compartment.

The proposed models broadly fall into five groups (see Table 1). One group contains single compartment models; the remaining four groups are categorized according to the anisotropy of the perfusion and diffusion compartments. For example, “anisotropic‐isotropic” refers to models with anisotropic perfusion compartment and isotropic diffusion compartment.

### Data Acquisition

DWI was performed on volunteers using a 3T Philips Achieva scanner with a 32‐channel cardiac coil. The study involved a cohort of nine healthy pregnant subjects with gestational age (GA) between 27 + 5 (weeks + days) and 38 + 0. For posterior placentas we found that the SNR was inadequate, due to the distance between the placenta and receiver coil. Therefore all subjects included in this study had an anterior placenta. Informed consent was obtained for all scans (REC number 14/LO/1169). All subjects were scanned in the supine position during free breathing. Medical records were reviewed after delivery, with eight subjects delivering infants with birth weight between the 7th and 95th percentiles. The birth details for one subject were not available, as they were lost to follow‐up. We implemented a previously published single‐shot spin echo EPI sequence which is optimized for reduced acoustic noise, peripheral nerve stimulation, and RF heating 25. Reduction of these effects is an important safety consideration during fetal scans. We developed a rich protocol spanning a wider range of *b*‐values and gradient directions than would typically be available, to identify the most expressive model that the signal potentially supports.

The three principal gradient directions were scanned at *b* = 15, 25, 80, 115, 206, 246, and 346 s mm^−2^, and eight directions were obtained at *b* = 40, 400, 1000, 2000 s mm^−2^. Six *b* = 0 images were also obtained. For all scans the *b* = 0 volumes were distributed throughout the acquisition. Other settings were as follows: TR = 3792 ms, TE = 132 ms, FOV = 300 × 300 × 44 mm^3^, 2.2 mm isotropic voxels, 20–25 contiguous slices, gradient duration = 0.0224 s, diffusion time = 0.0656 s. The slices were acquired in the axial direction (with respect to the mother) for seven subjects, and coronally for two subjects. For five scans the diffusion‐weighted images were obtained in ascending *b*‐value order (scan duration: 3 min 55 s). For the remaining four scans we used a protocol with interspersed high and low *b*‐value slices (scan duration: 4 min 1 s). The latter protocol aims to improve the suitability of the data for subsequent respiratory motion correction 26 in the future.

### Model Fitting

A placental region of interest (ROI) and uterine wall ROI were manually defined on the first *b* = 0 image. These ROIs are not segmentations of tissue types, which would be very difficult at this resolution, but rather correspond to broad anatomical areas. We fit 14 models (Table 1) voxel‐by‐voxel to the complete set (all *b*‐values) of DWI measurements within the masked regions. The model parameters were fit to the normalized DWI signal with maximum log‐likelihood estimation assuming Rician noise, as previously described 23, 27. Specifically, the log‐likelihood is

(1) $$\ln\hat{L}=\sum_{i=1}^{N}\left[\ln S_{i}-2\ln\sigma -\frac{S_{i}^{2}-{\tilde{S}}_{i}^{2}}{2\sigma^{2}}+\ln I_{0}\left(\frac{S_{i}{\tilde{S}}_{i}}{\sigma^{2}}\right)\right]$$

where ${\left\{{\tilde{S}}_{i}\right\}}_{i=1}^{N}$ are the measured signals, ${\left\{S_{i}\right\}}_{i=1}^{N}$ are the model predicted signals, *σ* is the standard deviation on the real and imaginary parts of the signal, and *I*_0_ is the modified Bessel function of the first kind. To fit models within this framework we require constraints on the parameter values. We constrained the parameters over a range of biologically plausible values (https://onlinelibrary.wiley.com/action/downloadSupplement?doi=10.1002%2Fmrm.27036&attachmentId=213714997). For multicompartment models, we constrain the diffusivities of the perfusion and diffusion compartments above and below 5 × 10^−3^ mm^2^ s^−1^, respectively. This is slightly higher than the diffusion coefficient of water at 37°C (3 × 10^−3^ mm^2^ s^−1^), because the diffusion compartment potentially includes some slow perfusion of maternal blood. Following 28 we estimate the SNR accounting for Rician noise bias to be around 20 on average. We therefore chose to fix the SNR at 20 for all model fits.

To visualize broad trends in the DWI signal across *b*‐values we also fit the diffusion tensor model (using the non‐linear fitting option in Camino 29) to each of the eight‐gradient shells (*b* = 40, 400, 1000, 2000 s mm^−2^) individually. We hence computed mean diffusivity (MD) and fractional anisotropy (FA) parameter maps specific to each of these *b*‐value shells.

### Model Selection

We use standard statistical model‐selection techniques to determine which models are best supported by the DWI signal in the placenta. Specifically, in each voxel, and for each model, we calculated the Bayesian information criterion (BIC),

(2) $$\;\text{BIC}=-2\ln\hat{L}+k\ln n,$$

where $\ln\hat{L}$ is the maximized value of the log‐likelihood function given in Eq. (1) ( $\ln\hat{L}$ is proportional to the fitting error), *k* is the number of model parameters (Table 1), and *n* is the number of observations (i.e., the total number of diffusion‐weighted images ‐ 59). The model with the lowest BIC value best explains the data, i.e., provides the best trade off between model complexity and goodness of fit. Additionally the strength of preference between a pair of models can be assessed with $\Delta \text{BIC}={\;\text{BIC}}_{1}-{\;\text{BIC}}_{2}$, where BIC_*i*_ is the BIC for model *i*. A ΔBIC of 10 or more implies “decisive” preference for the model with lower BIC 30.

## RESULTS

**Figure 2:**
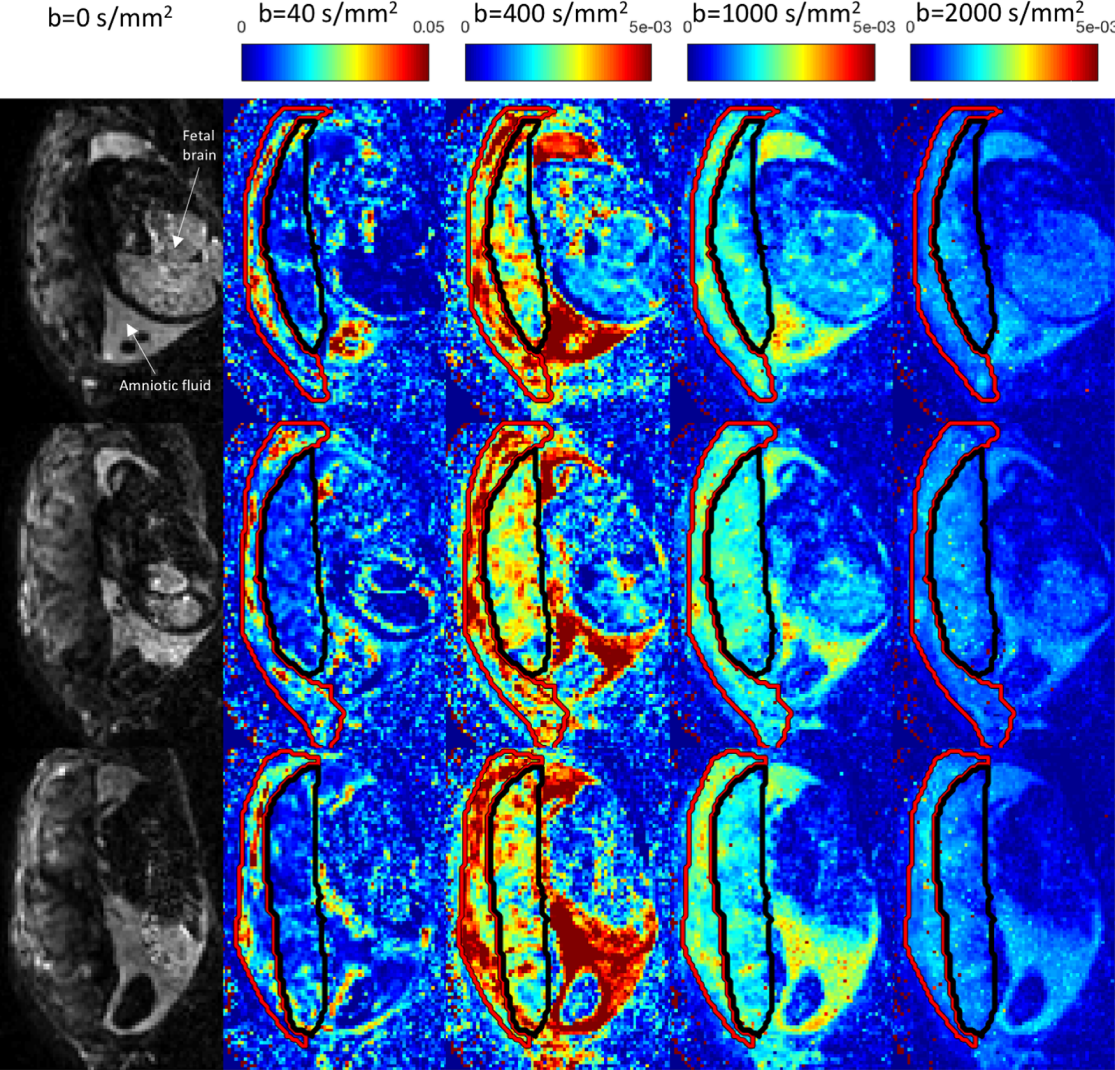
Mean diffusivity maps for three slices of a single placental diffusion‐weighted MRI scan. Gestational age: 35 + 6 (weeks + days). The columns *b* = 40 s mm^−2^ to *b* = 2000 s mm^−2^ show the mean diffusivities derived from a diffusion tensor fit only to the images at that nonzero *b*‐value and *b* = 0. Red and black outline the uterine wall and placenta ROIs respectively. Color represents mean diffusivity in mm^2^ s^−1^ (note that the scale is a factor of 10 higher for the *b* = 40 s mm^−2^ maps).

**Figure 3:**
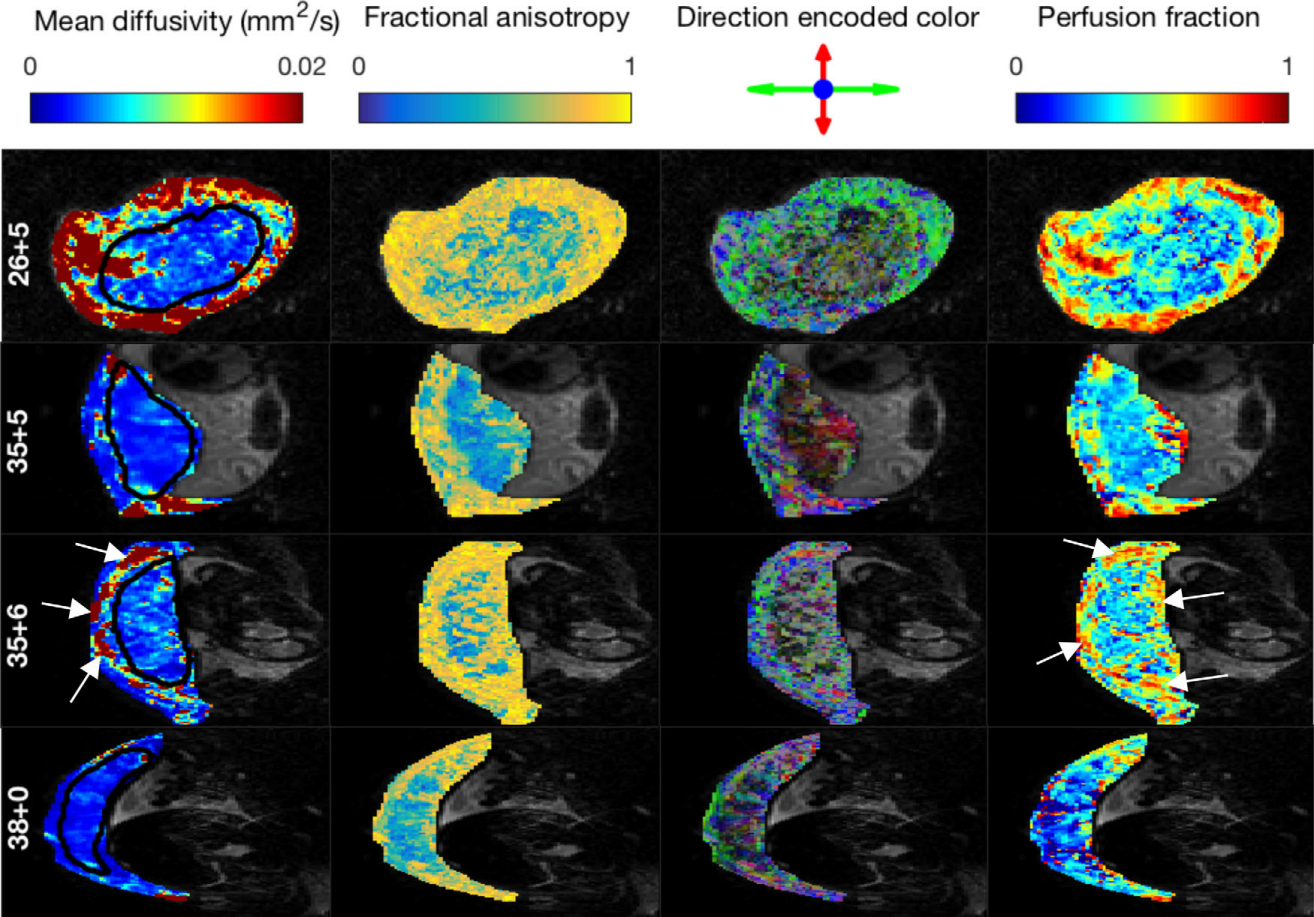
Parameter maps derived from diffusion tensor and ball‐ball model fits. Each row displays maps for a single slice from one subject, labeled by gestational age (weeks + days). Slices are displayed in the EPI acquisition plane, corresponding to the coronal plane (row 1) and axial plane (remaining rows). Arrows in row 3 highlight areas of high diffusivity and high perfusion at the boundary of the placenta. https://onlinelibrary.wiley.com/action/downloadSupplement?doi=10.1002%2Fmrm.27036&attachmentId=213714997 is the complete version of this figure, containing these maps for all subjects.

**Figure 4:**
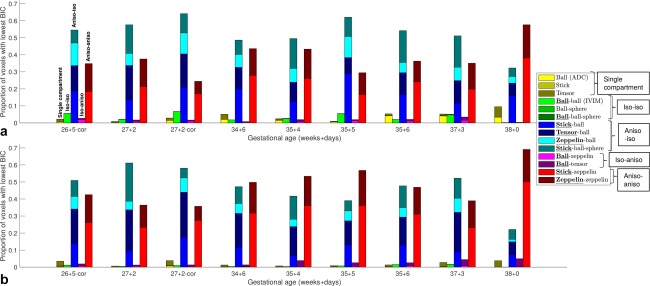
Model selection results across all subjects. Bar plots showing the proportion of voxels where each model had the lowest Bayesian information criterion for nine placental scans. Subjects are labeled by gestational age, with “‐cor” indicating that the placenta was scanned coronally. The perfusion model compartment is emphasized in the legend text. **a**: Placenta ROI. **b**: Uterine wall ROI.

**Figure 5:**
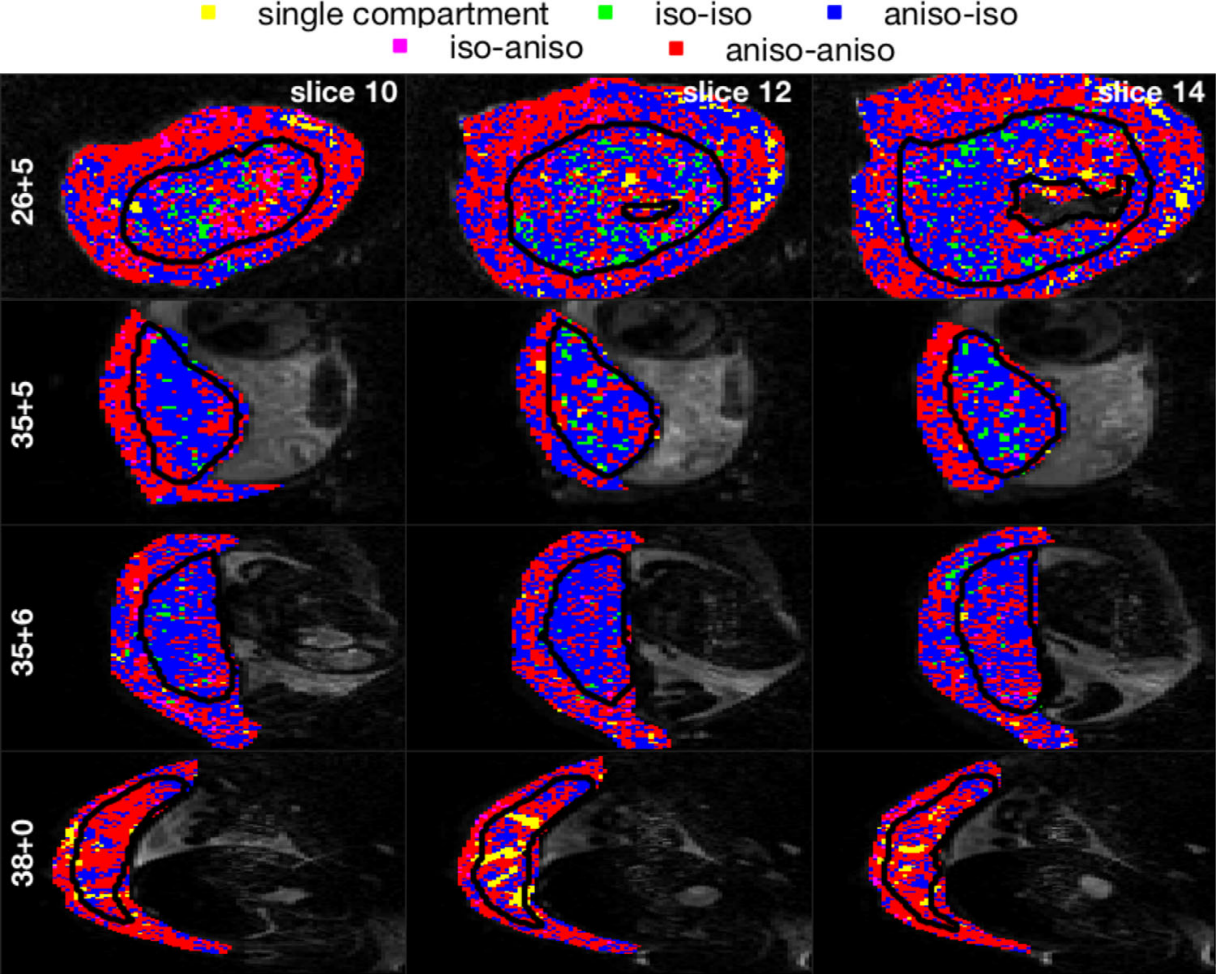
Mapping the spatial pattern of model selection results. Each row displays three slices for a single subject, labeled by gestational age (weeks + days). Voxels are colored according to the category of the model with the lowest Bayesian information criterion in that voxel. Models are labeled according to the isotropy of the perfusion and diffusion compartments, respectively, for example “aniso‐iso” refers to models with anisotropic perfusion compartment and isotropic diffusion compartment. Slices are displayed in the EPI acquisition plane (coronal plane for row 1, axial plane for other rows). https://onlinelibrary.wiley.com/action/downloadSupplement?doi=10.1002%2Fmrm.27036&attachmentId=213714997 is the complete version of this figure, containing these maps for all subjects.

**Figure 6:**
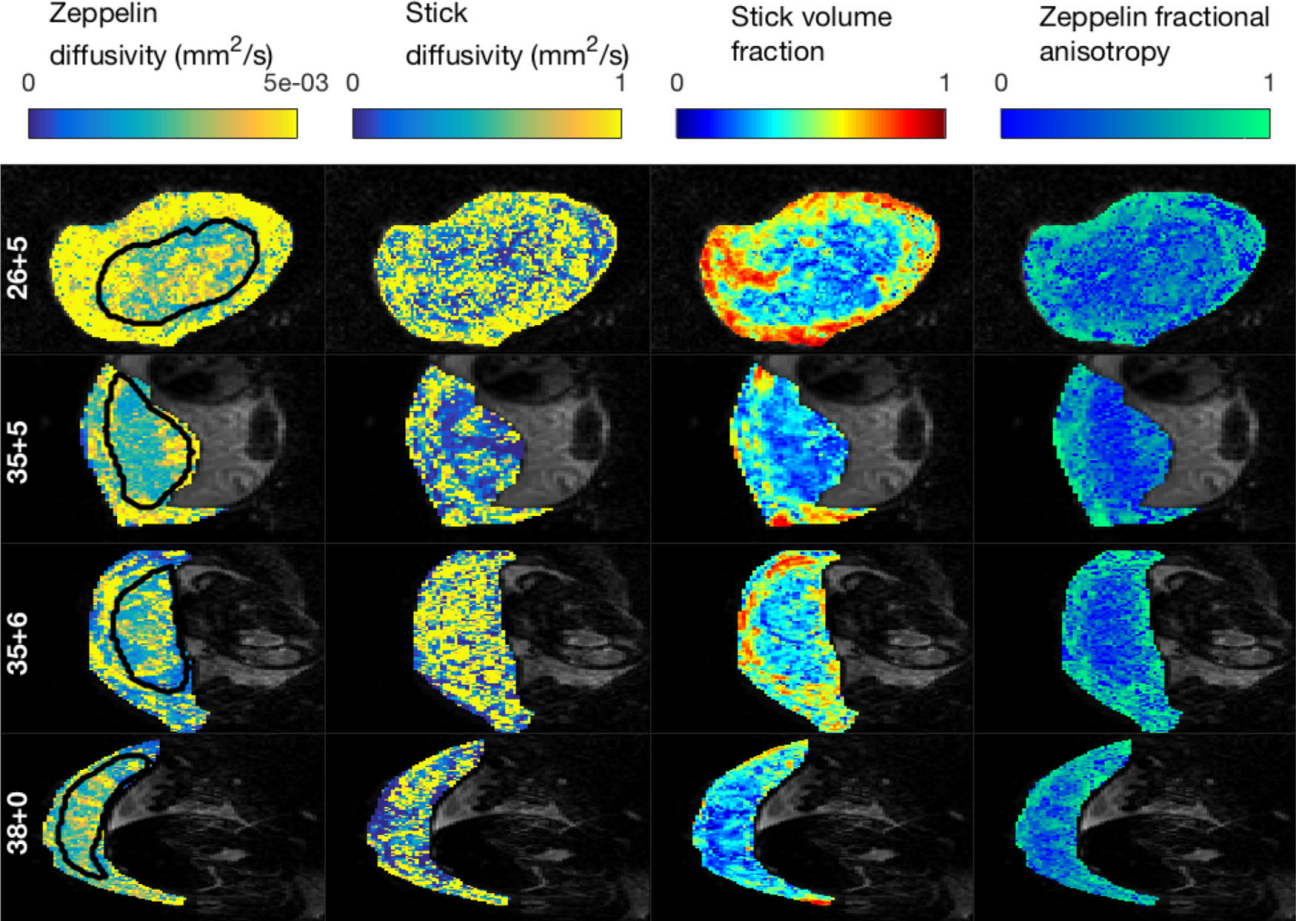
Parameter maps derived from stick‐zeppelin model. Each row displays maps for a single axial slice from one subject, labeled by gestational age (weeks + days). Slices are displayed in the EPI acquisition plane (coronal plane for row 1, axial plane for other rows). https://onlinelibrary.wiley.com/action/downloadSupplement?doi=10.1002%2Fmrm.27036&attachmentId=213714997 is the complete version of this figure, containing these maps for all subjects.

Figures 2 and 3 present standard DTI and ball‐ball parameter maps over the nine subjects. We then examine model selection results across all 14 models (Figs. 4 and 5), showing that the classical models are outperformed by the new models. Next, we display parameter maps (Fig. 6) and a bootstrapping analysis for a single model which performed very well in model selection. Finally, we comment on the relationship between derived parameters and GA for a range of models.

The first column in Figure 2 displays three slices from a *b* = 0 volume for a single subject, scanned axially. The placenta, uterine wall, fetal brain, and amniotic fluid can all be distinguished. The remaining columns show the MD maps specific to the *b* = 40, 400, 1000, and 2000 s mm^−2^ shells. Contrast across different areas of the maps is clearly visible. For example, at lower *b* values (*b* = 40 s mm^−2^ and *b* = 400 s mm^−2^ shells) the MD is significantly higher in the uterine wall ROI than the placental ROI, potentially reflecting high levels of perfusion in maternal arteries and veins. There is also contrast across different areas of the placenta. Notably, some contrast remains even at higher (1000, 2000 s mm^−2^) *b*‐values. For example, at *b* = 2000 s mm^−2^ the inner (i.e., fetal) placenta has a noticeably lower MD than the outer placenta and amniotic fluid.

Figure 3 shows DTI (fit to all *b*‐values) and ball‐ball parameter maps for four subjects, and https://onlinelibrary.wiley.com/action/downloadSupplement?doi=10.1002%2Fmrm.27036&attachmentId=213714997 displays the same data for all nine subjects. These maps are consistent with the known physiology. For example MD maps, in common with single‐shell maps in Figure 2, show patches of higher diffusivity along the uterine wall and chorionic plate. These patches correspond with areas of higher perfusion fraction in ball‐ball maps (Fig. 3 and https://onlinelibrary.wiley.com/action/downloadSupplement?doi=10.1002%2Fmrm.27036&attachmentId=213714997, 4th column), suggesting that they reflect areas with a high volume of blood flowing through blood vessels. FA (Fig. 3 and https://onlinelibrary.wiley.com/action/downloadSupplement?doi=10.1002%2Fmrm.27036&attachmentId=213714997, 2nd column) and direction encoded color (Fig. 3 and https://onlinelibrary.wiley.com/action/downloadSupplement?doi=10.1002%2Fmrm.27036&attachmentId=213714997, 3rd column) maps clearly show that there are high levels of anisotropy, particularly in the regions of tissue at the boundary of the placenta. This is likely due to fibrous cells in the uterine wall and chorionic plate, as well as the coherent orientation of vasculature in these areas. These observations are consistent across axial and coronal scanning planes.

Figure 4 shows the proportion of voxels where each model has the lowest BIC value, and hence reveals broad trends in model preference. We plotted this proportion for the placenta and uterine wall ROIs separately, and grouped models depending on the isotropy of the perfusion and diffusion compartments. We present the same data in https://onlinelibrary.wiley.com/action/downloadSupplement?doi=10.1002%2Fmrm.27036&attachmentId=213714997, and additionally highlight the top three models for each scan. For the placenta and uterine wall ROIs, and in all subjects, the signal is best explained by two‐compartment, non‐restriction models incorporating some level of anisotropy (“anisotropic IVIM” models). The dominant model categories are anisotropic‐isotropic and anisotropic‐anisotropic. In other words, the models which best explain the DWI signal have an anisotropic perfusion compartment, and either an isotropic or anisotropic diffusion compartment. For the placenta and uterine wall ROIs, and for most subjects, stick‐zeppelin and zeppelin‐zeppelin models are in the top three models ranked by the proportion of voxels with the lowest BIC value. Additionally, in voxels where a model other than stick‐zeppelin or zeppelin‐zeppelin best explained the data, they are still close to the best model (https://onlinelibrary.wiley.com/action/downloadSupplement?doi=10.1002%2Fmrm.27036&attachmentId=213714997). Stick‐ball and tensor‐ball models are also preferred in a significant proportion of voxels, with the former better in the placenta and the latter in the uterine wall. Of the three models with a restricted compartment stick‐ball‐sphere is by far the best, having the lowest BIC in 5–22% of voxels depending on the subject and ROI. There is no noticeable difference in model preference between coronal and axial scans.

Figure 5 and https://onlinelibrary.wiley.com/action/downloadSupplement?doi=10.1002%2Fmrm.27036&attachmentId=213714997 map the category of the model with the lowest BIC, which largely reflects spatial patterns in the isotropy of the diffusion compartment. In accordance with Figure 4, anisotropic IVIM models almost always explain the signal best, although different model categories tend to be favored in different areas. Models with isotropic diffusion compartment generally perform better within the placenta, whereas models with anisotropic diffusion compartment are best at the boundaries of the placenta, i.e., within the uterine wall and chorionic plate.

Figure 6 and https://onlinelibrary.wiley.com/action/downloadSupplement?doi=10.1002%2Fmrm.27036&attachmentId=213714997 show parameter maps for the stick‐zeppelin model, since this performed consistently well in model selection, both in the placenta and the uterine wall. They reveal additional information, specific to perfusion and diffusion compartments, that can be accessed using anisotropic IVIM models. Zeppelin diffusivity maps appear to reveal cotyledon structure for some subjects (e.g., Fig. 6 1st column, final row). Stick diffusivity maps (2nd column) show much variability, with very high parameter values in many voxels. The 4th column shows the FA of the diffusion compartment, the results mirror those in Figure 5 and https://onlinelibrary.wiley.com/action/downloadSupplement?doi=10.1002%2Fmrm.27036&attachmentId=213714997: there is mostly low FA within the placenta, and high FA in the uterine wall and chorionic plate.

We performed a bootstrapping analysis to estimate the standard deviations of stick‐zeppelin model parameters (https://onlinelibrary.wiley.com/action/downloadSupplement?doi=10.1002%2Fmrm.27036&attachmentId=213714997). The ratio of parameter values to parameter standard deviations is typically around 10. Parameter standard deviations are much lower than the difference in values between regions; we can therefore confidently infer contrast across these regions in stick‐zeppelin parameter maps.

Finally, we made an initial exploration into the relationship between model‐derived parameters and GA. https://onlinelibrary.wiley.com/action/downloadSupplement?doi=10.1002%2Fmrm.27036&attachmentId=213714997 plots the DTI‐derived MD against GA, and https://onlinelibrary.wiley.com/action/downloadSupplement?doi=10.1002%2Fmrm.27036&attachmentId=213714997 plots the perfusion fraction for three models against GA. Both parameters decreased across gestation, and interestingly these correlations were higher in the uterine wall than the placenta. Encouragingly, although we emphasize that we only have nine cross‐sectional samples, these trends are consistent with previous reports for the ADC 31 and ball‐ball perfusion fraction 32.

## DISCUSSION

This article demonstrates 3T DWI of the human placenta and uterine wall using a multishell, multidirectional imaging protocol. We fit a range of microstructural models to the DWI signal, and assess which models best explain the data. Encouragingly, even though the achievable resolution is limited as we did not correct for respiratory motion, we observe consistent patterns in parameters and model selection statistics across a cohort of nine subjects. These trends can be summarized as follows:

1. Anisotropic IVIM models describe the in vivo human placenta diffusion MRI signal better than ADC, ball‐ball (i.e., IVIM) and DTI models.
2. The fast‐attenuating signal component is anisotropic in nearly all voxels.
3. The slow‐attenuating signal component is anisotropic in 20–70% of voxels, depending on ROI and subject. This anisotropy is most prevalent in the uterine wall and chorionic plate.

As we discuss in the following sections, these patterns are consistent with the structure and physiology of the placenta and surrounding tissue. This suggests that the DWI signal is sensitive to anatomically linked microstructural and microvascular features.

### Anisotropic IVIM

The ball‐ball (i.e., IVIM) model 20 assumes that blood is flowing in capillaries with uniformly distributed orientation giving rise to isotropic signal attenuation. Recently, there have been multiple extensions proposed which model the effect of more coherent microvascular orientation, by considering anisotropy in the perfusion (and sometimes diffusion) signal. These methods have been applied in kidney 33, 34, heart 35, skeletal muscle 36, brain 37, and cancer 24 imaging.

Here, we fit a range of multicompartment models which contain an anisotropic perfusion or diffusion compartment (or both). Figure 4 and https://onlinelibrary.wiley.com/action/downloadSupplement?doi=10.1002%2Fmrm.27036&attachmentId=213714997 show that there is anisotropy in the fast‐attenuating signal component for the vast majority of voxels in the placenta and surrounding tissue. Models with stick compartments—the most anisotropic possible—also show consistently good performance (Fig. 4). These observations match the interpretation that there is a coherent orientation of the vasculature. This may be necessary in order to facilitate the transport of large volumes of maternal and fetal blood into and out of the placenta (e.g., Fig. 1). The data shows anisotropy in the slow‐attenuating signal component that varies—both spatially and across subjects (https://onlinelibrary.wiley.com/action/downloadSupplement?doi=10.1002%2Fmrm.27036&attachmentId=213714997, 4th column). For most scans, we observed anisotropy in the slow‐attenuating signal component within the uterine wall and chorionic plate. This is consistent with an assignment of the slow‐attenuating signal component to water in tissue, as tissue in these areas contains highly ordered smooth muscle and fibrous cell types. The low anisotropy in the slow‐attenuating signal component within placental areas may arise from large maternal blood pools in intervillous space exhibiting isotropic diffusion during flow; it could also arise from the fact that, at the voxel scale, tissue consisting of highly convoluted villi has less coherent orientation.

### Areas with Diffusion Restriction

The diffusion signal persists at relatively high *b*‐values (Fig. 2), and restriction models fit the DWI signal best in 5–22% of voxels, depending on tissue type and subject (Fig. 4, https://onlinelibrary.wiley.com/action/downloadSupplement?doi=10.1002%2Fmrm.27036&attachmentId=213714997). These effects are correlated: areas where signal persists at high *b*‐values often correspond with stick‐ball‐sphere being the preferred model (https://onlinelibrary.wiley.com/action/downloadSupplement?doi=10.1002%2Fmrm.27036&attachmentId=213714997, circled areas in 1st and 2nd columns). https://onlinelibrary.wiley.com/action/downloadSupplement?doi=10.1002%2Fmrm.27036&attachmentId=213714997 also shows sphere volume fraction maps; notably there are many areas within the placenta with zero sphere fraction, suggesting no detectable restriction (https://onlinelibrary.wiley.com/action/downloadSupplement?doi=10.1002%2Fmrm.27036&attachmentId=213714997, arrows). This may correspond to areas dominated by maternal blood within intervillous space. We also plotted sphere radius maps (https://onlinelibrary.wiley.com/action/downloadSupplement?doi=10.1002%2Fmrm.27036&attachmentId=213714997, 4th column) that reveal areas with low sphere radius and non‐zero sphere volume fraction (https://onlinelibrary.wiley.com/action/downloadSupplement?doi=10.1002%2Fmrm.27036&attachmentId=213714997, circled areas in 3rd and 4th columns), suggesting diffusion restriction. This is consistent with higher cellularity in these areas, although we emphasize that, despite our use of a spherical restriction model, outer cell membranes are not the only structures that can cause restriction.

Our initial observations suggest some evidence for restricted diffusion, but it does not appear to be a dominant effect. This interpretation is consistent with the placental anatomy, which contains large areas of intervillous space where restriction is unlikely to occur. Quantification of restriction may have applications in early assessment of placental abnormalities; for example, the invasion of trophoblast cells into the uterine wall plays an important role in the normal remodeling of spiral arteries during early pregnancy 38, 39, 40. Future acquisition protocols with higher *b*‐values and SNR may provide better access to this compartment.

### Future Work

The sensitivity of DWI to microstructure shows promise for early assessment of placental abnormalities. Diagnosis of FGR currently relies on Doppler ultrasound of the umbilical cord and uterine artery, along with fetal biometry 11, 12. Pre‐eclampsia is diagnosed upon presentation of maternal symptoms 13. In both cases, there has already been substantial inhibition of placental function at the point of diagnosis. Thus far, the utility of monitoring placental health with DWI has been investigated using ADC 31, 41, 42, ball‐ball (i.e., IVIM) 32, 43, 44, 45, 46 and DTI 47 models. The ADC model may be useful as a diagnostic tool for FGR, but the evidence is limited to a single study 41. Ball‐ball is the most frequently used model in the placenta, and has been fairly successful in relating DWI signals to pathologies, with the perfusion fraction (an estimate of the relative volume of flowing blood) being significantly lower in FGR 43, 45, 46 and early onset pre‐eclampsia 32. The DWI models presented in this paper show potential for improved characterization of placental microstructure, although there are a number of challenges still to be addressed.

A clear area for further development is data acquisition. For example, scanning at higher resolution would bring many advantages, such as sensitivity to higher spatial frequency changes in tissue microstructure. Clinically limited acquisition times are another important consideration, especially since DWI is often part of a larger multimodal scan. Therefore development of techniques which offer speed ups, such as interleaving of *b*‐values 26 and multiband acceleration 48, 49, are beneficial. Once a particular model or set of models is chosen, we can also use experimental design optimization, e.g., as in 50, to reduce acquisition time and increase sensitivity to key parameters.

Better image post‐processing also offers further improvements in placental microstructure imaging. In this paper, we perform no motion correction, but assume alignment across DWI volumes. This undoubtedly affects the visual quality of DWI parameter maps 51, limiting our ability to image small‐scale structures, such as spiral arteries. Motion correction in the placenta is a difficult and little studied problem, and requires consideration of non‐rigid motion (both inter‐slice and inter‐volume) 15. In the future, we aim to develop algorithms for motion correction in conjunction with protocols interspersing high and low *b*‐value slices, as used for four subjects here, since these improve registration between diffusion weighted volumes.

In this paper, we assessed a broad range of biophysical models, but future work will concentrate on models which quantify placental microstructure and microcirculation well. Stick‐zeppelin and zeppelin‐zeppelin show the most promise, due to their consistently high‐ranking across the placenta and uterine wall in model selection analysis. Zeppelin‐zeppelin is the more general model, since a zeppelin compartment can capture isotropic diffusion when the parallel and perpendicular diffusivities are equal (unlike the strict anisotropy of the stick compartment). It also has more parameters, and generally explains the placental data better when it is not the best model (https://onlinelibrary.wiley.com/action/downloadSupplement?doi=10.1002%2Fmrm.27036&attachmentId=213714997). These reasons lead us to prefer zeppelin‐zeppelin for rich multishell, multidirection acquisition protocols, such as the one presented here. For sparser imaging protocols stick‐zeppelin would be easier to fit to the data, having three fewer parameters, and may be a more robust choice. We observed that stick diffusivity estimates are very variable with high values in many voxels (https://onlinelibrary.wiley.com/action/downloadSupplement?doi=10.1002%2Fmrm.27036&attachmentId=213714997, 2nd column). This is likely due to the difficulty in accurately quantifying fast diffusion, since measurements of fast‐attenuating voxels are highly sensitive to noise. Any real anatomical variation in these maps would be very difficult to distinguish from the high variance due to the aforementioned effect. In future studies it may be better to fix this parameter to a physiologically reasonable value.

Our models assume that relaxivity values are constant across compartments, but it is highly likely that T_1_, T_2_, and $T_{2}^{*}$ values vary across compartments, e.g., due to oxygenation levels of blood and tissue. This would cause a weighting of the inferred volume fractions by the corresponding relaxivities. Future studies could address this by using complementary relaxivity and diffusivity measurements to improve placental microstructure characterization, as in Ref. 52. This is an important consideration within the placenta as there is a gradient in the oxygenation of maternal blood from spiral arteries to decidual veins, which affects the $T_{2}^{*}$ value.

Finally, the key area for future work is to translate the findings in this paper on suitable models for placental diffusion into biomedical applications of quantitative imaging. Although it was not the main purpose of this study, we made an initial assessment into the extent to which model‐derived parameters reflect changes in microstructure throughout gestation. The observation of model parameter changes with GA motivates future work investigating and quantifying this dependence more directly. Longitudinal studies would give a more direct assessment of the relationship between model‐derived parameters and GA. Compartment models offer potential improvements over earlier studies of diffusion parameters against GA, by modeling specific biophysical features. https://onlinelibrary.wiley.com/action/downloadSupplement?doi=10.1002%2Fmrm.27036&attachmentId=213714997 shows that MD, both in the placenta and uterine wall, is the parameter that correlates best with GA. However, this observation does not have an obvious biophysical interpretation, as MD averages over perfusion and diffusion effects. https://onlinelibrary.wiley.com/action/downloadSupplement?doi=10.1002%2Fmrm.27036&attachmentId=213714997 reveals that the large decrease in MD may be due to the compounding of separate effects: a reduction in the volume of flowing blood, and a reduction in diffusivity. In other situations, strong effects that act in opposite directions on MD can cancel out, but compartment models can still reveal them. The clear next step after a wider study quantifying microstructural changes across gestation is to extend to pathological placentas. By scanning subjects with pregnancy complications such as FGR and pre‐eclampsia, we will investigate parameter values in these placentas, and therefore assess the efficacy of model‐based DWI for quantifying placental pathologies.

## CONCLUSIONS

In this paper, we demonstrate that anisotropic IVIM models explain the in vivo human placenta DWI signal better than ADC, ball‐ball (i.e., IVIM) and DTI models when using a rich, multishell, multidirectional protocol. These models can extract quantitative values related to the diffusivity, anisotropy, and relative fractions of the fast‐ and slow‐attenuating components of the diffusion signal. Parameters derived from model fits could potentially capture changes in placental microstructure across gestation. Initial observations were consistent with the previous literature—diffusivity and perfusion fraction decreased with GA. The identification of models which best explain the placental diffusion signal will underpin future development of scanning protocols. We anticipate that these optimized protocols will further elucidate which model‐derived parameters best quantify variation in placental microstructure. This approach naturally extends to pathological placentas, where we will aim to assess which image‐derived biomarkers capture the differences between normal and pathological tissue.

## Supporting information


**Fig. S1**. Parameter maps derived from DTI and ball‐ball model fits. Each row displays maps for a single slice from one subject, labelled by GA. Slices are displayed in the EPI acquisition plane, corresponding to the coronal plane (row 1 and 3) and axial plane (remaining rows). Arrows in row 7 highlight areas of high diffusivity and high perfusion at the boundary of the placenta.
**Fig. S2**. Stick‐zeppelin and zeppelin‐zeppelin are close to the best model in most voxels. Cumulative histograms of the difference between stick‐zeppelin and zeppelin‐zeppelin BICs, and the lowest BIC across all models in that voxel. A) Placenta ROI, B) uterine wall ROI.
**Fig. S3**. Mapping the spatial pattern of model selection results. Each row displays three slices for a single subject, labelled by GA. Voxels are coloured according to the category of the model with the lowest BIC in that voxel. Models are labelled according to the isotropy of the perfusion and diffusion compartments respectively, for example “aniso‐iso” refers to models with anisotropic perfusion compartment and isotropic diffusion compartment. Slices are displayed in the EPI acquisition plane (coronal plane for rows 1 and 3, axial plane for other rows).
**Fig. S4**. Parameter maps derived from stick‐zeppelin model. Each row displays maps for a single axial slice from one subject, labelled by GA. Slices are displayed in the EPI acquisition plane (coronal plane for rows 1 and 3, axial plane for other rows).
**Fig. S5**. Standard deviation of stick‐zeppelin parameters from bootstrapping analysis. The data (i.e. 59 diffusion‐weighted images) was resampled with replacement 100 times, and the stick‐zeppelin model was fit to each resampled dataset. This enabled estimation of the standard deviation of stick‐zeppelin model parameters (note that the color scales are 5 times lower than those in Fig. 6 and Supporting Fig. S4). Each row displays maps for a single axial slice from one subject, labelled by GA. Slices are displayed in the EPI acquisition plane (coronal plane for rows 1 and 3, axial plane for other rows).
**Fig. S6**. MD decreases as a function of GA. Scatter plot showing the median value of the MD within two ROIs against GA, A) Placenta ROI, B) uterine wall ROI.
**Fig. S7**. Perfusion fraction decreases as a function of GA. As Supporting Figure S6 except plotting the median value of the perfusion fraction for three models.
**Fig. S8**. Stick‐ball‐sphere parameter maps. Each row displays maps for a single axial slice from one subject, labelled by GA. Slices are displayed in the acquisition plane. The second column shows the MD calculated from a DTI fit only to the images at b=0 and b=2000 s mm^– 2^. In the 5th row an area where stick‐ball‐sphere was the preferred model and the signal persisted at high b‐values is circled, and arrows show areas with zero sphere volume fraction. In the 7th row an area with low sphere radius and non‐zero sphere volume fraction is circled.
**Table S1**. Constraints on parameters when fitting models to the DWI signal. *D* denotes a diffusion coefficient which was constrained to reasonable values for water diffusion. *D_*v*_* was constrained at a much higher value, and can hence model water flowing within vascular structures. *D_*v*_* has a reduced lower threshold in models where the perfusion and diffusion compartments are combined (i.e. single compartment models and ball‐sphere). There is one additional constraint for all models: the volume fractions for all compartments sum to 1.
**Table S2**. Proportion of voxels in the placenta ROI where each model had the lowest BIC value. The three models with the highest proportions for each scan are highlighted. Subjects are labelled by GA, with “‐cor” indicating that the placenta was scanned coronally.
**Table S3**. Proportion of voxels in the uterine wall ROI where each model had the lowest BIC value. As Supporting Table S2, but for the uterine wall ROI.
