# Supplementary material for: Placenta microstructure and microcirculation imaging with diffusion MRI
Source: Magn Reson Med. 2017 Dec 11;80(2):756–66. doi: 10.1002/mrm.27036 (PMC5947291; doi:10.1002/mrm.27036)
Supplement: Supplementary file 1 — Fig. S1. Parameter maps derived from DTI and ball‐ball model fits. Each row displays maps for a single slice from one subject, labelled by GA. Slices are displayed in the EPI acquisition plane, corresponding to the coronal plane (row 1 and 3) and axial plane (remaining rows). Arrows in row 7 highlight areas of high diffusivity and high perfusion at the boundary of the placenta. Fig. S2. Stick‐zeppelin and zeppelin‐zeppelin are close to the best model in most voxels. Cumulative histograms of the difference between stick‐zeppelin and zeppelin‐zeppelin BICs, and the lowest BIC across all models in that voxel. A) Placenta ROI, B) uterine wall ROI. Fig. S3. Mapping the spatial pattern of model selection results. Each row displays three slices for a single subject, labelled by GA. Voxels are coloured according to the category of the model with the lowest BIC in that voxel. Models are labelled according to the isotropy of the perfusion and diffusion compartments respectively, for example “aniso‐iso” refers to models with anisotropic perfusion compartment and isotropic diffusion compartment. Slices are displayed in the EPI acquisition plane (coronal plane for rows 1 and 3, axial plane for other rows). Fig. S4. Parameter maps derived from stick‐zeppelin model. Each row displays maps for a single axial slice from one subject, labelled by GA. Slices are displayed in the EPI acquisition plane (coronal plane for rows 1 and 3, axial plane for other rows). Fig. S5. Standard deviation of stick‐zeppelin parameters from bootstrapping analysis. The data (i.e. 59 diffusion‐weighted images) was resampled with replacement 100 times, and the stick‐zeppelin model was fit to each resampled dataset. This enabled estimation of the standard deviation of stick‐zeppelin model parameters (note that the color scales are 5 times lower than those in Fig. 6 and Supporting Fig. S4). Each row displays maps for a single axial slice from one subject, labelled by GA. Slices are displayed in the EPI acquis [file MRM-80-756-s001.pdf]

Parameter	Description	Constraints
D	diffusivity	$0.01 \times 10^{-3} \leq D \leq 5 \times 10^{-3}\,\mathrm{mm^2\,s^{-1}}$
D_v (combined perfusion and diffusion)	pseudo-diffusion coefficient	$0.01 \times 10^{-3} \leq D_v \leq 1000 \times 10^{-3}\,\mathrm{mm^2\,s^{-1}}$
D_v (all other models)	pseudo-diffusion coefficient	$5 \times 10^{-3} \leq D_v \leq 1000 \times 10^{-3}\,\mathrm{mm^2\,s^{-1}}$
D_{sphere}	diffusivity within impermeable sphere	$0.01 \times 10^{-3} \leq D_{sphere} \leq 1000 \times 10^{-3}\,\mathrm{mm^2\,s^{-1}}$
$D^{\parallel}$	diffusivity in principle direction	$0.01 \times 10^{-3} \leq D^{\parallel} \leq 5 \times 10^{-3}\,\mathrm{mm^2\,s^{-1}}$
$D^{\perp}$	diffusivity in perpendicular directions	$0.01 \times 10^{-3} \leq D^{\perp} \leq 5 \times 10^{-3}\,\mathrm{mm^2\,s^{-1}}$
$D_v^{\parallel}$	pseudo-diffusion in principle direction	$5 \times 10^{-3} \leq D_v^{\parallel} \leq 1000 \times 10^{-3}\,\mathrm{mm^2\,s^{-1}}$
$D_{v1}^{\perp}, D_{v2}^{\perp}, D_v^{\perp}$	pseudo-diffusion in perpendicular directions	$0.01 \times 10^{-3} \leq D_{v1}^{\perp}, D_{v2}^{\perp}, D_v^{\perp} \leq 1000 \times 10^{-3}\,\mathrm{mm^2\,s^{-1}}$
r	sphere radius	$0.001 \leq r \leq 0.02\,\mathrm{mm}$
f_v	perfusion volume fraction	$0 \leq f_v \leq 1$
f_{sphere}	restriction volume fraction	$0 \leq f_{sphere} \leq 1$
ϕ, θ, ψ	angles defining tissue orientation	$-100 \leq \phi, \theta, \psi \leq 100\,\mathrm{rad}$
ϕ_v, θ_v, ψ_v	angles defining vascular orientation	$-100 \leq \phi_v, \theta_v, \psi_v \leq 100\,\mathrm{rad}$

Supporting Table S1: **Constraints on parameters when fitting models to the DWI signal.** D denotes a diffusion coefficient which was constrained to reasonable values for water diffusion. D_v was constrained at a much higher value, and can hence model water flowing within vascular structures. D_v has a reduced lower threshold in models where the perfusion and diffusion compartments are combined (i.e. single compartment models and ball-sphere). There is one additional constraint for all models: the volume fractions for all compartments sum to 1.

	26.71-cor	27.29	27.29-cor	34.86	35.57	35.71	35.86	37.43	38
ADC	0.01	0.00	0.02	0.02	0.02	0.01	0.04	0.04	0.03
Stick	0.00	0.00	0.00	0.00	0.00	0.00	0.00	0.00	0.00
Tensor	0.02	0.01	0.01	0.03	0.01	0.00	0.01	0.01	0.06
IVIM	0.06	0.02	0.07	0.02	0.02	0.05	0.02	0.05	0.00
Stick-ball	**0.19**	0.14	**0.21**	**0.20**	0.13	**0.29**	**0.17**	0.12	**0.16**
Tensor-ball	0.15	**0.20**	**0.20**	0.13	0.11	0.09	0.13	0.13	0.08
Zeppelin-ball	0.13	0.07	0.12	0.08	0.08	0.12	0.06	0.08	0.04
Ball-zeppelin	0.02	0.01	0.01	0.00	0.01	0.01	0.01	0.02	0.00
Ball-tensor	0.01	0.01	0.01	0.00	0.01	0.01	0.01	0.02	0.00
Stick-zeppelin	**0.19**	**0.21**	**0.17**	**0.28**	**0.26**	**0.17**	**0.24**	**0.20**	**0.38**
Zeppelin-zeppelin	**0.16**	0.16	0.07	**0.16**	**0.17**	**0.13**	0.12	**0.15**	**0.19**
Ball-sphere	0.00	0.00	0.00	0.00	0.00	0.00	0.00	0.00	0.00
Ball-ball-sphere	0.00	0.00	0.00	0.00	0.00	0.00	0.00	0.00	0.00
Stick-ball-sphere	0.08	**0.17**	0.11	0.08	**0.18**	0.11	**0.19**	**0.18**	0.05

Supporting Table S2: **Proportion of voxels in the placenta ROI where each model had the lowest BIC value.**

The three models with the highest proportions for each scan are highlighted. Subjects are labelled by GA, with "-cor" indicating that the placenta was scanned coronally.

	26.71-cor	27.29	27.29-cor	34.86	35.57	35.71	35.86	37.43	38
ADC	0.00	0.00	0.01	0.00	0.00	0.00	0.00	0.00	0.00
Stick	0.00	0.00	0.00	0.00	0.00	0.00	0.00	0.00	0.00
Tensor	0.03	0.01	0.03	0.01	0.01	0.01	0.01	0.03	0.04
IVIM	0.01	0.00	0.01	0.00	0.00	0.01	0.01	0.02	0.00
Stick-ball	0.14	0.09	**0.17**	0.12	0.07	0.13	0.13	0.09	0.07
Tensor-ball	**0.20**	**0.24**	**0.26**	**0.20**	**0.17**	**0.14**	**0.16**	**0.23**	**0.08**
Zeppelin-ball	0.07	0.05	0.08	0.06	0.04	0.06	0.06	0.08	0.01
Ball-zeppelin	0.01	0.01	0.00	0.00	0.00	0.01	0.01	0.01	0.00
Ball-tensor	0.01	0.01	0.01	0.01	0.03	0.02	0.02	0.04	0.05
Stick-zeppelin	**0.26**	**0.23**	**0.27**	**0.32**	**0.36**	**0.36**	**0.31**	**0.23**	**0.50**
Zeppelin-zeppelin	**0.16**	0.13	0.08	**0.18**	**0.17**	**0.21**	**0.16**	**0.16**	**0.19**
Ball-sphere	0.00	0.00	0.00	0.00	0.00	0.00	0.00	0.00	0.00
Ball-ball-sphere	0.00	0.00	0.00	0.00	0.00	0.00	0.00	0.00	0.00
Stick-ball-sphere	0.09	**0.22**	0.06	0.10	0.14	0.06	0.13	0.12	0.06

Supporting Table S3: **Proportion of voxels in the uterine wall ROI where each model had the lowest BIC value.**

As Supporting Table S2, but for the uterine wall ROI.

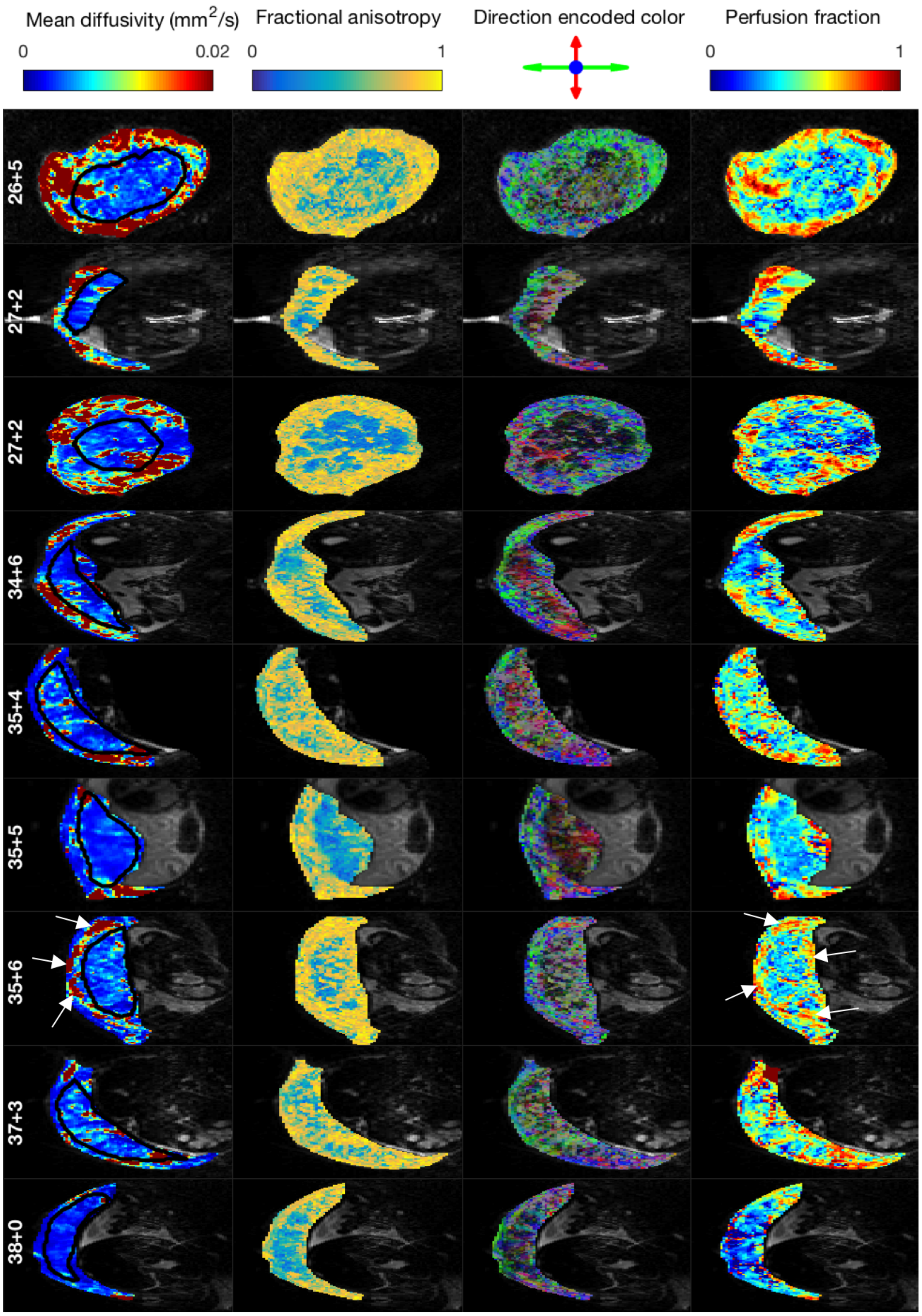

Supporting Figure S1: **Parameter maps derived from DTI and ball-ball model fits.** Each row displays maps for a single slice from one subject, labelled by GA. Slices are displayed in the EPI acquisition plane, corresponding to the coronal plane (row 1 and 3) and axial plane (remaining rows). Arrows in row 7 highlight areas of high diffusivity and high perfusion at the boundary of the placenta.

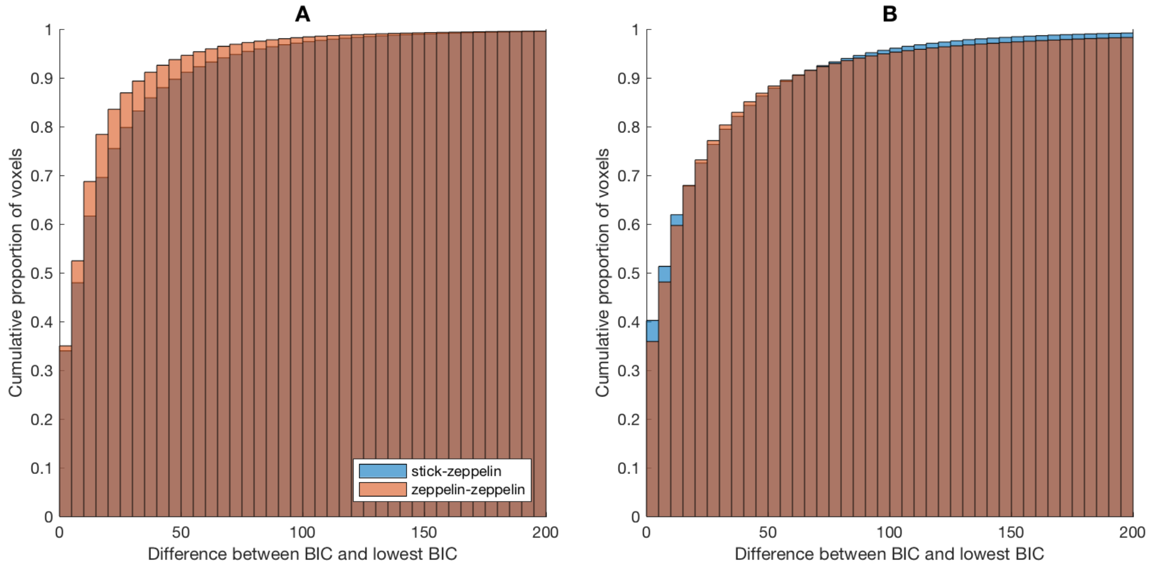

Supporting Figure S2: **Stick-zeppelin and zeppelin-zeppelin are close to the best model in most voxels.**

Cumulative histograms of the difference between stick-zeppelin and zeppelin-zeppelin BICs, and the lowest BIC across all models in that voxel. A) Placenta ROI, B) uterine wall ROI.

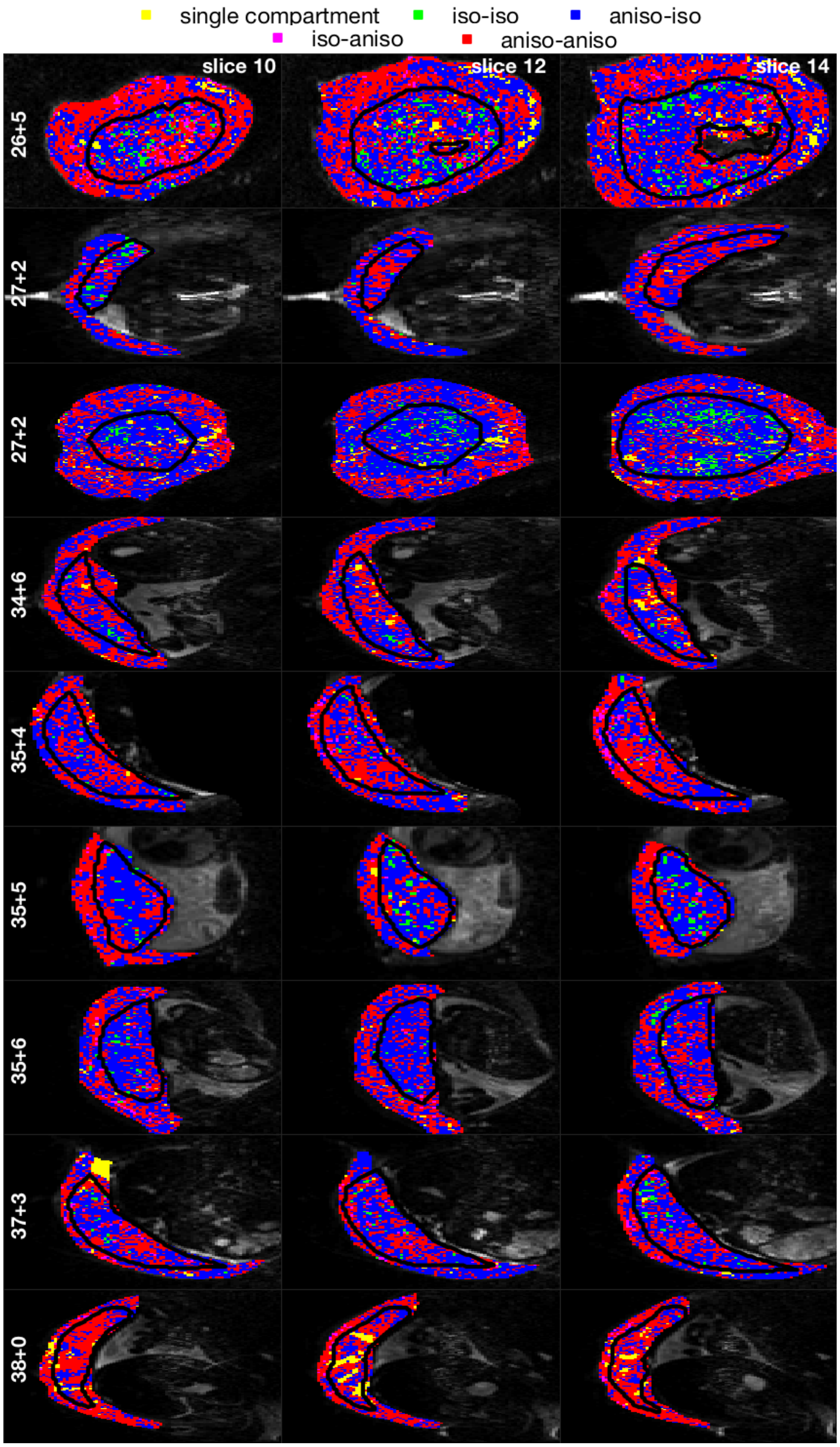

Supporting Figure S3: **Mapping the spatial pattern of model selection results.**

Each row displays three slices for a single subject, labelled by GA. Voxels are coloured according to the category of the model with the lowest BIC in that voxel. Models are labelled according to the isotropy of the perfusion and diffusion compartments respectively, for example "aniso-iso" refers to models with anisotropic perfusion compartment and isotropic diffusion compartment. Slices are displayed in the EPI acquisition plane (coronal plane for rows 1 and 3, axial plane for other rows).

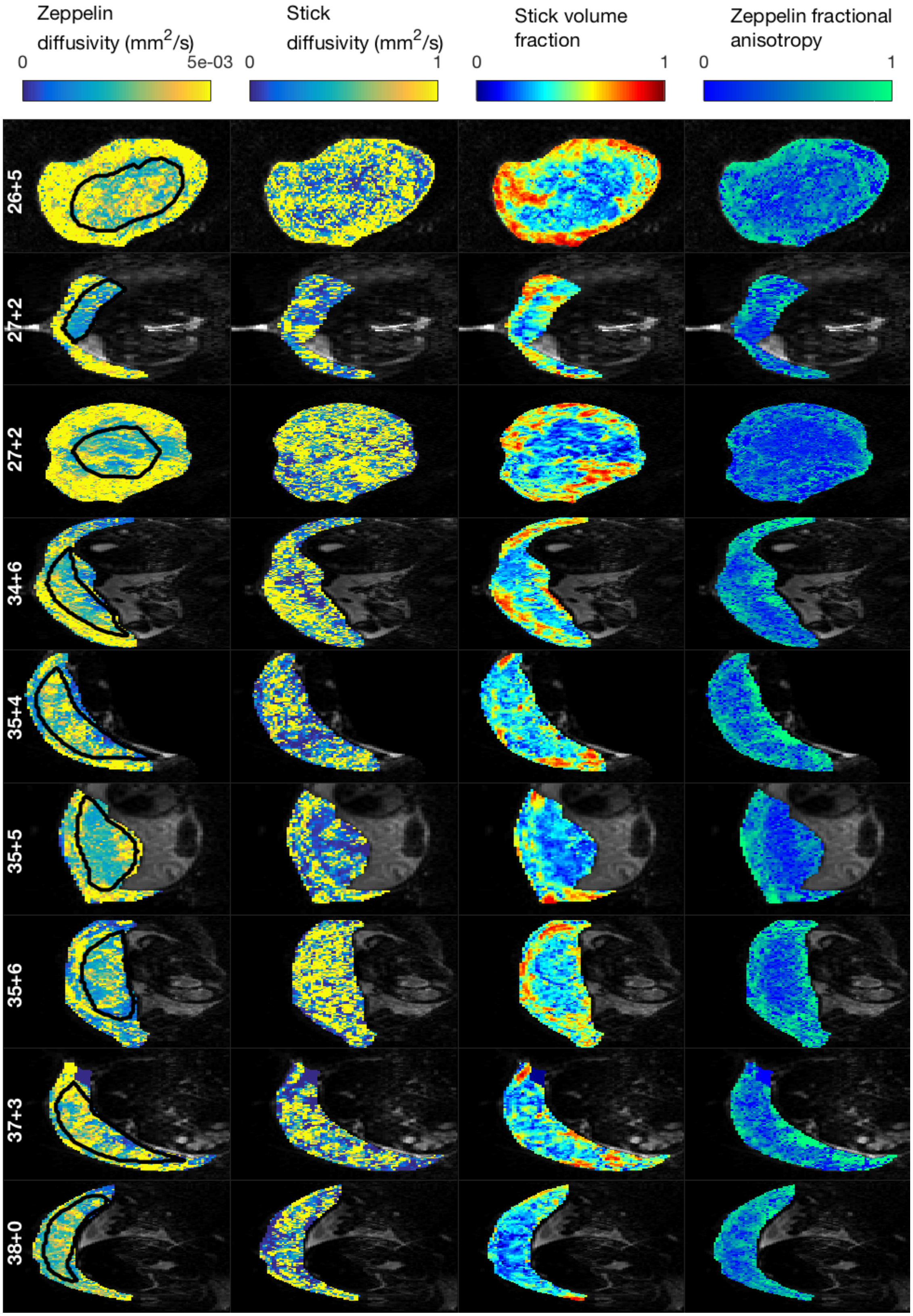

Supporting Figure S4: **Parameter maps derived from stick-zeppelin model.**
Each row displays maps for a single axial slice from one subject, labelled by GA. Slices are displayed in the EPI acquisition plane (coronal plane for rows 1 and 3, axial plane for other rows).

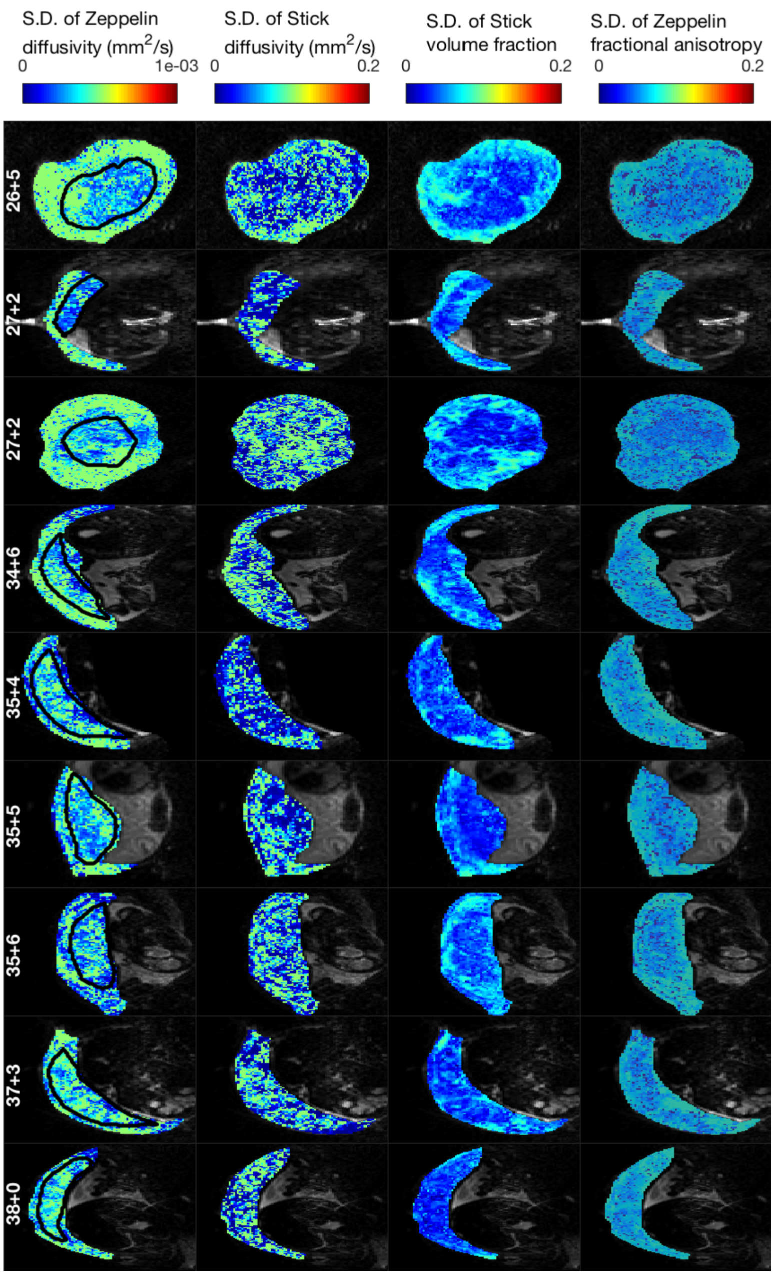

Supporting Figure S5: **Standard deviation of stick-zeppelin parameters from bootstrapping analysis.**

The data (i.e. 59 diffusion-weighted images) was resampled with replacement 100 times, and the stick-zeppelin model was fit to each resampled dataset. This enabled estimation of the standard deviation of stick-zeppelin model parameters (note that the color scales are 5 times lower than those in Figure 6 and Supporting Figure S4). Each row displays maps for a single axial slice from one subject, labelled by GA. Slices are displayed in the EPI acquisition plane (coronal plane for rows 1 and 3, axial plane for other rows).

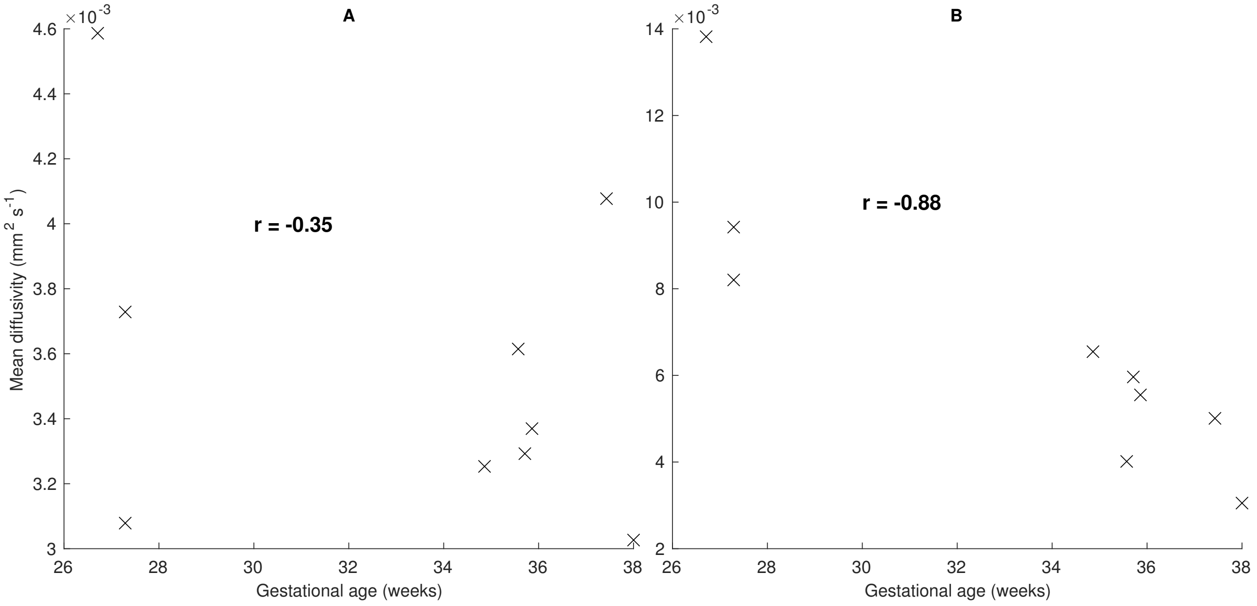

Supporting Figure S6: **MD decreases as a function of GA.**

Scatter plot showing the median value of the MD within two ROIs against GA, A) Placenta ROI, B) uterine wall ROI.

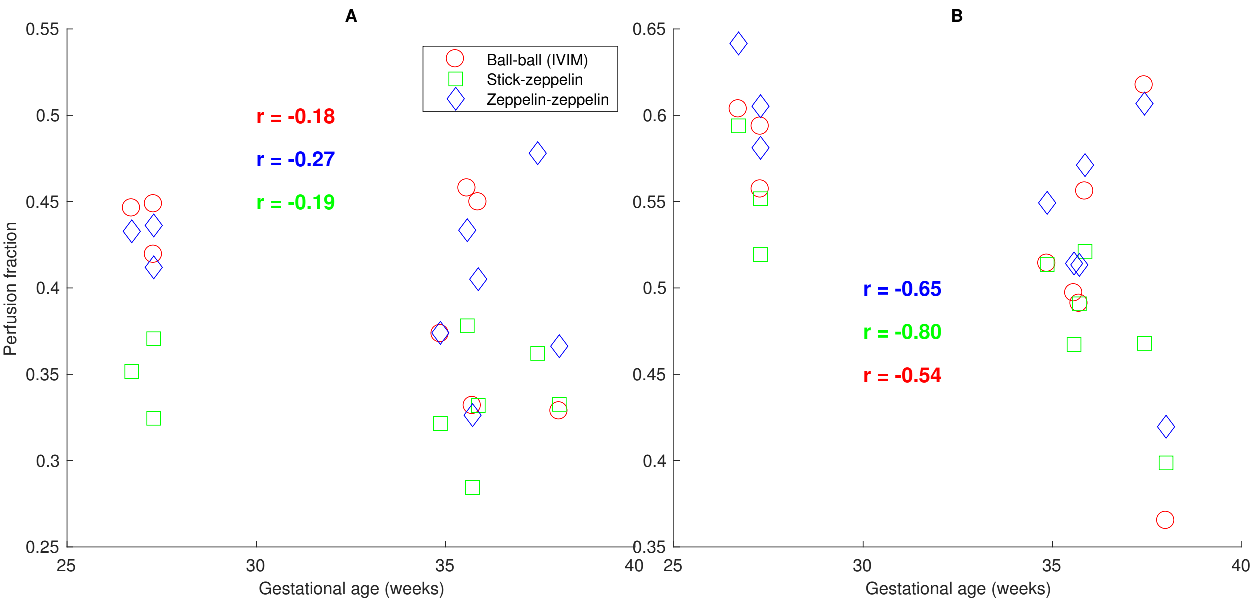

Supporting Figure S7: **Perfusion fraction decreases as a function of GA.**

As Supporting Figure S6 except plotting the median value of the perfusion fraction for three models.

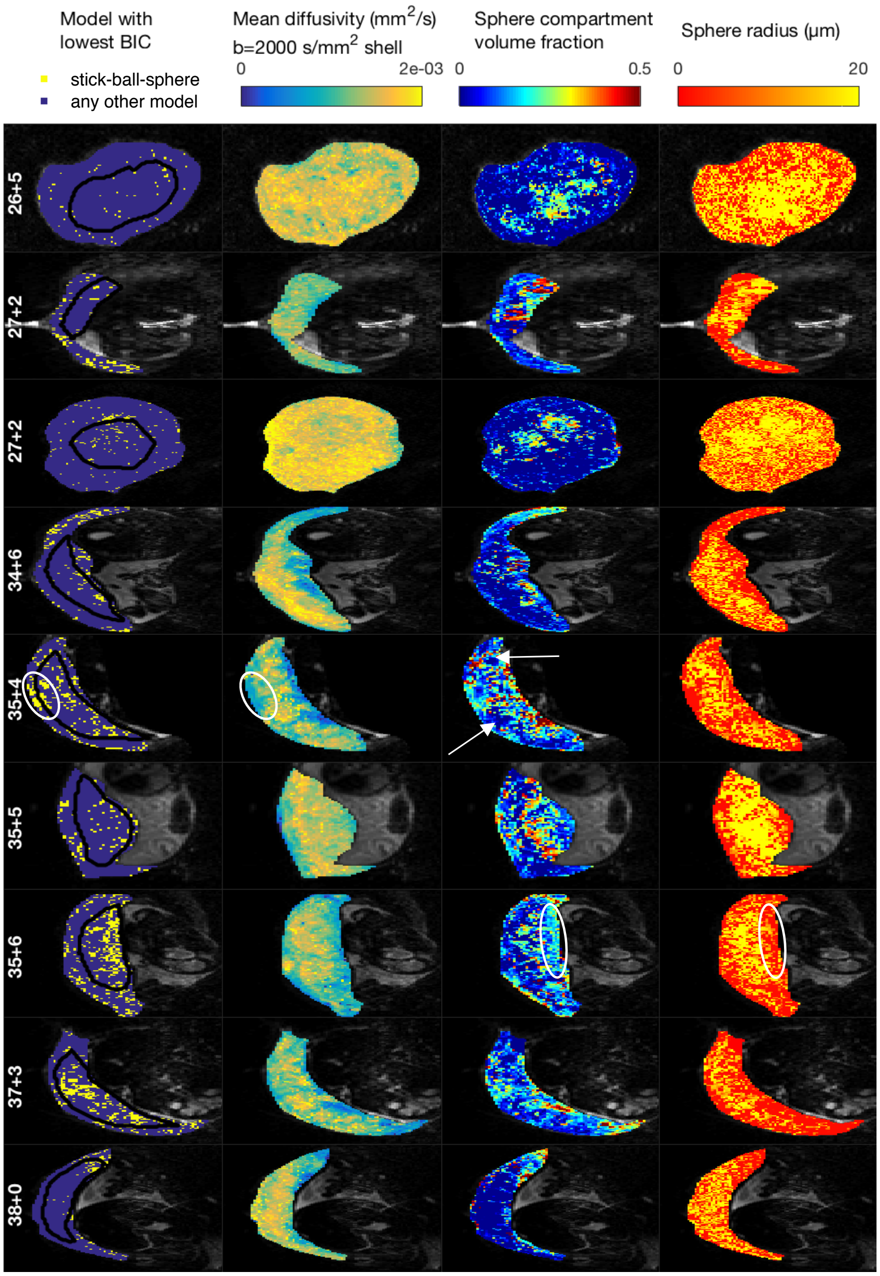

Supporting Figure S8: **Stick-ball-sphere parameter maps.**

Each row displays maps for a single axial slice from one subject, labelled by GA. Slices are displayed in the acquisition plane. The second column shows the MD calculated from a DTI fit only to the images at b=0 and b=2000 s mm^{-2}. In the 5th row an area where stick-ball-sphere was the preferred model and the signal persisted at high b-values is circled, and arrows show areas with zero sphere volume fraction. In the 7th row an area with low sphere radius and non-zero sphere volume fraction is circled.
